# Phrenology

**Published:** 1860-04

**Authors:** V. H. Taliaferro

**Affiliations:** Atlanta, Georgia


Vol. V.] APRIL, 1860. [No. 8.

### ORIGINAL COMMUNICATIONS.

### ARTICLE I.

*Phrenology.* By V. H. Taliaferro, M. D., of Atlanta, Georgia.

In the January or February issue of the Atlanta *Medical and Surgical Journal* there appears an article by Joseph W. Clift, upon the above subject, which takes in review our editorial article in the *Medical and Literary Weekly* of July 2d, upon "Temperament *vs.* Phrenology." He sets out with the assertion that, "The writer seems never to have studied Phrenology with an unprejudiced mind, and labors under a misconception of its teachings." Were this charge true, our views in relation to it would doubtless have been more in accordance with those advanced by our critic and reviewer. In reference to this charge of handling that, we understood not, we will simply state that, we have been the student of Ancient and Modern Phrenologists; the notorious "*Water-Cure Doctors*," Fowler and Wells, included; and if we are not much mistaken, these great *Hydropathic* lights stand at the head of American Phrenology. Perchance these false dogmas would have led us far in the dark, but for the sound practical truths of Dunglison, Dalton, Draper, Paine and Carpenter, whose wise teachings hold up in the clear sunlight of science the palpable errors and deceptions of Phrenological teachings. With these lights, and the guidance in our anatomical demonstrations of that great man, Granville Sharp Patterson, we saw no evidences of truth in Phrenology. Yes, sir, we have studied Phrenology with the same spirit which guides us in the investigation of any and all scientific questions, and most candidly believe it a "speculative theory," co-equal in its ridiculous folly and error with clarvoyance, mesmerism and witchcraft.

In the paragraph following the one in which we "seem" not to have studied, &c., our reviewer says: "Phrenology teaches that as we possess a pair of hands, eyes, ears, &c., so in like manner are our mental faculties double, &c." We have no question that were it not unmistakeably perceptible that but one head belongs to every man, it would be stubbornly claimed that as he has two hands and two feet, "so in like manner" must he have two heads. And furthermore, says our reviewer: "His allusion to the "Flat Heads" is equally unfortunate for his argument. Because, development of certain faculties of the mind is always followed by increased nutrition, and certain portions of the brain are enlarged faster than surrounding organs, it does not justify the conclusion he seems to have arrived at, that we, by creating an abnormal growth, could change the faculties." Abnormal means unnatural, or a departure from a given rule or regularity. Now if an organ be unusually or unnaturally large, it is abnormally developed, whether by a natural or mechanical process. The arm of the blacksmith is abnormally large, and yet its strength is proportionate to its size, and were it in our power to develop muscular structure without the exercise, it would be equally powerful.

Were Phrenology true—every faculty of the mind having its distinct and separate locality upon the brain, and the strength and power of each faculty judged by its corresponding cranial protuberance—then, most assuredly, would the "flat heads" be deficient in all their nobler traits, from the fact of mechanical obstructions to the growth of the organs, characterizing them, while the more lateral and posterior organs of the brain would be abnormally developed, and in a corresponding proportion, abnormally active and powerful. But the Phrenologist would answer these objections to his favored so-called science, by saying that if an organ or any number of organs were prevented, by any mechanical means, from development, that the activity of such organs would not be interfered with, because of a corresponding increase in the development of the brain elsewhere. Truly is this a flimsy argument for Phrenology. For instance, were we to make pressure upon the organs of benevolence and veneration, and thereby prevent their growth, other sections of the brain, having different and distinct functions to perform, could not take upon themselves the double office of supplying these deficiencies, because their functions are separately distinct. But holding, on the other hand, that mind can only be made manifest by the action of the brain as a whole, then these difficulties are easily solved, and we are at no loss to understand why it is that the characteristic traits of the Indian are the same, no matter in what shape he may grow his cranium.

Phrenologists, since the day of Gaul and Spurzheim, have based their theory upon the idea of the plurality of the mental faculties, having severally and separately their distinct localities in the brain—each one of these distinct localities having, as it were, independent functions, and being known, as to their size, strength and power, by developments upon the cranium. These then should accordingly be their only guide, but in order the more effectually to elude the force of all arguments against it, they are now claiming as within the *broad scope* [ha! ha!] of Phrenological science, those general characteristics which belong to different temperaments, different races, and different animals. As well might the "two hands," "two feet," gout included, be termed Phrenological. Physical organization gives peculiar character to temperaments, races and animals. An Irishman is an Irishman, no matter under what sun he claims his birth. An American is the same in general characteristics, though born and reared upon European soil. When Phrenologists, by examination of craniums, will give us the different temperaments and peculiar characteristics of different races, then we will begin to admit that there is truth in Phrenology; but, instead of this, they, as frequently as otherwise, fail to point out the most prominent faculties of the mind, or represent the weakest faculties as being the most prominent and powerful. A reputable and far-famed champion of Phrenology, upon examination of my own cranium, told me that my *musical* talent was unusually developed—that I could, with little trouble, perform well upon any instrument, and *sing any song*—and, indeed, that music was the great delight of my nature, and that this talent was inherited from my mother, who, without doubt, was exceedingly musical. This announcement very naturally created no little mirth among my acquaintances, knowing, as they did, my inability, from childhood, to be able to sing or whistle correctly the most simple song, or play a tune upon any instrument, notwithstanding my more musically inclined father, hitched me regularly to every singing school, fiddle school, or other musical institution which might happen along; and what is still very remarkable, my mother never was known to sing a song so that it could be recognized. This is no isolated case—they are numerous.

We assert upon physiological authority and the science of accumulated ages, that plurality of mental manifestation, as belonging severally and separately to distinct and particular parts of the brain, is unestablished in the slightest degree. Gaul and his confederates most signally failed in their dissections of the brain, to elucidate their theory, but, instead, added confusion to uncertainty. The National Institute of France, influenced by the pretensions of Drs. Gaul and Spurzheim deputed five of its most learned and scientific members to carefully dissect the human brain, in connection with the authors of this new and astounding theory, to ascertain the grounds, if any, for its establishment, and give a

"clear and precise abstract of it." The result was, that the report of this committee, far from confirming the views of Gaul, proved a most insurmountable barrier to the adoption of his views, and to day, Phrenology is almost wholly discarded by scientific men and authors. Dunglison, Carpenter, Dalton, Draper, Paine, all, discard it upon the ground of a false basis, while the world-renowned quacks—"Water Cure" —Fowler & Wells, lead the van of seekers after Phrenological wonders.

Were Phrenology true, the National Institute Committee would certainly have found, as they expected, some anatomical divisions or limits, to separate parts of the brain, having distinct and different functions. But, alas, for the beautiful theory of Gaul and Spurzheim, no such divisions or limits could be detected, and yet how exactly precise, even to the hundredth part of an inch, is checked off upon the craniums the different faculties of the mind. The layer of gray matter which occupies the surface of the brain, and which ramifies with its beautifully folding convolutions, is continuous throughout each principal portion of the brain.

In this gray matter is supposed to reside the active functions of the brain, bearing the same relation to the other structures of the brain that the ganglions do to the nerve. Now, in nervous ganglia having separate functions, anatomical divisions are always traced; but the surface of the brain we find to be one continuous and compact pulpy mass, having not the minutest trace of dividing lines in its structure. We would as much expect separate distinct functions of the brain traceable by divisions as in nervous ganglia.

Cruvellhier, in his anatomy, says: "The depth of the convolutions varies from ten to fourteen lines, but it is extremely variable in different individuals; moreover, there are, perhaps, not two convolutions, nor two parts of the same convolution, which correspond in thickness in the same brain."

"It would be undoubtedly curious to describe minutely all the convolutions. Vesalius, who appears to have entertained the idea of so doing, likened the appearance of the surface of the brain to those irregular forms which are traced by unskilful painters in delineating clouds. Vicqd' Azyr made an unsuccessful attempt to elucidate this subject. Gaul and Spurzheim, who were interested in giving a minute description of each convolution, abandoned the task. I have myself attempted, and so has Rolando, to describe and name some of them." Thus do we perceive, from this standard anatonist, the impossibility of describing these convolutions, upon the mere superficial surfaces of which, according to Phrenology, are seated separately the organs of the brain. What a brazen absurdity, then, the idea of mapping out upon these variable Convolutions, the upper surfaces of which only can be explored, so *precisely accurate*, every faculty of the brain separately and distinctly; while indeed many of these convolutions never present themselves upon the outer surface of the brain, but lie buried beneath other and larger folds. One simple physiological and anatomical question successfully refutes the whole doctrine of phrenology, viz: In what part of the brain structure is seated, mainly its great function? *in this gray matter, which makes up the entire surface of the brain.* Is this entire surface subject to external exploration? *it is not*, and there is the end to the whole of it.

The pleurality of the mental faculties must be admitted; but we regard them as the result of the action of all parts of the brain, conjointly and in unison; and in this opinion we are sustained by every physiological and anatomical evidence bearing upon the subject. The manifestations of the mind are subject to many controlling circumstances, and which are not necessarily dependent upon any change in the organization of the brain. Though from any circumstances, a faculty of the mind be weakened or destroyed, *no corresponding change can be detected in its organ, as recognised by Phrenologists.* This is incompatible with all known physiological laws. "How decided an alteration in the disposition is produced by emasculation," and yet without the least change in the organ called amativeness. Now, it is well known that no nerve or tissue of the body loses its function without an evident and decided change in its structure.—"Two animals can scarcely be more different from each other than an ox or a bull," and yet though the psychical manifestations are so changed, the cerebellum* maintains its natural development and proportions. Prof. Dunglison in his Cyclopædia of Practical Medicine, says: "Cretins† in whom the cerebellum is defective, display more or less idiotism or defect of intellect, but no corresponding deficiency in the sexual instincts, which on the contrary often exists in such unhappy beings in the greatest intensity, and impels them to furious excess. Again, injuries of the posterior part of the head are observed to be followed by stupor and loss of memory, indicating the function of the cerebellum to be connected with the exercise of the mental faculties rather than animal propensities." This is a stubborn fact for Phrenology, and one within itself sufficient to stamp it for all time to come, as unreliable and untruthful.

We would call upon our reviewer for refuting *physiological facts*—we do not want speculation. When we remember that the convolutions of the brain are from ten to fourteen lines in depth, and therefore the edges only subject to external exploration, beside other equally strong facts, we are irresistibly forced to the conclusion that, not only is it a "speculative theory," but that its teachings are worthy the profoundest contempt of scientific men.

But still there comes a more serious objection to Phrenology as a science, than has yet been given. All the mental faculties have been located upon that portion of the brain accessible to external exploration. In saying *all* we mean *all*—every one—not a faculty but has its corresponding *accessible* organ. Now, we emphatically assert, that the *entire sur-*

---

*According to Gaul and Spurzheim, the animal instincts all have their seat in the cerebellum.

†This is the name given to deformed and wretched beings met with in Pyrenees, Berne and upper Gascony. In France they are called Caputs.

*face of the base of the brain*, including the greater part of the cerebellum is inaccessible to *phrenological fingers tout ensemble*. Not only so, but the inner surfaces of the convolutions, those of the great longitudinal fissure, as well as the surfaces of the fissure of sylvius which divides the cerebrum into an anterior and middle lobe are equally inaccessible.

These hidden surfaces make up at least two-thirds of the whole surface of the brain, and we would, in all sincerity, ask if it is reasonable to suppose that it has no agency in mental manifestations, because it is inaccessible and cannot be made to fit phrenological theory.

Putting aside all scientific facts, Phrenology can not be sustained by outside evidences, as the collection and comparison of crania of individuals of different mental traits.

Prof. Dunglison, whose physiological research and complation surpasses any of his day, says: "If for example we should examine a hundred monomaniacs, in all of whom certain feelings and propensities has been developed even to morbid excess, and it should be discovered by a person competent to form a judgment on this subject, that no evidence displays itself in the cranidocopy of so many individuals tending to support the doctriue, we should hold that it ought in all fairness to be abandoned. Some hundreds and even thousands of such persons have passed a part of their lives under the inspection of M. Esquiral, who possesses most extensive resources for elucidating almost every subject connected with the history of mental diseases, and has neglected no inquiry which could further the attainment of that object. The result of his observation will be allowed to be of some weight on the discussion of this question, in which the appeal is principally to facts of the precise description of those with which he has been chiefly conversant. At his establishment at Ivry he has a large assemblage of crania and casts from the heads of lunatics, collected by him during the long course of his attendance at the Salpetriere and at the Royal Hospital at Charenton, which is under his superintendence. While inspecting this collection, the writer of the present article was assured by M. Esquiral, that the testimony of his experience is entirely adverse to the doctrine of the Phrenologists—it has convinced him that there is no foundation whatever, in the facts for the system of correspondences which they lay down between certain measurements of the existence of certain mental endowments. There are few if any individuals in Europe whose sphere of observation has been so extensive as that of M. Esquiral."

Phrenology a science! One would doubtless be convinced (?) by the skillful and wonderful manipulations of such mesmerising luminaries as we have recently had among us, who not only mesmerise their subjects, but call in play any mental faculty, by touching its corresponding cranial development. We have as yet seen only human crania examined, but we suppose that *well educated* Phrenological fingers would very readily decide as to the amiability—kicking or running qualities of a horse, give their color, number of spots, their durability, &c., as well as give the pedigree of chickens, their capacity for laying, &c., also localizing the *fives*, *tens*, *twenty's*, *&c.*, within *iron safes* by the corresponding developments, depressions, and such like *incontestible* (*?*) evidences.

Our reviewer winds up his defence of Phrenology with the following questions: 1st. "If the brain continually acts as a unit, how and *why* can we exercise several faculties at one and the same time?" 2d. "And why is there such a thing known as a *monomaniac?*" 3d. "And why is Phrenology false, when it teaches that the intellectual faculties of the negro are so small that he is better fitted for manual labor than intellectual effort?" 4th. "And why do we instinctively judge of a stranger's intellect by the shape and general conformation of his head?"

To question first we *deny* that several faculties of the mind can be called into exercise at one and the same time, else we might translate Eurypides, solve one of Euclid's most difficult problems, and laugh and talk with our friends of past occurrences, "*at one and the same time.*" To question second we would answer, that "such a thing as a monomaniac" is known; because a man may lose his capacity to control his mind upon a particular subject, which may occur from a variety of circumstances. *Monomania* cannot be established by pathological evidences in the brain, which in case Phrenology were true, there would be no difficulty in doing.—In answer to question third, we would state that we were not aware the negro's capacity was judged of by the weight or bulk of his head. If so we would expect a few Websters looming up from among the unambitious thick-lipped species. We think we could measure some *whappers* in our locality. To question fourth we would reply that, *we* did not know that *strangers'* intellects were known by the shape of their heads, and were not apprised before that any body else knew it.

---

